# Risk Stratification Based on a Pattern of Immunometabolic Host Factors Is Superior to Body Mass Index—Based Prediction of COVID-19-Associated Respiratory Failure

**DOI:** 10.3390/nu14204280

**Published:** 2022-10-13

**Authors:** David M. Cordas dos Santos, Lian Liu, Melvin Gerisch, Johannes C. Hellmuth, Michael von Bergwelt-Baildon, Wolfgang G. Kunz, Sebastian Theurich

**Affiliations:** 1 Department of Medicine III, University Hospital, LMU Munich, 81377 Munich, Germany; 2 Cancer and Immunometabolism Research Group, Gene Center LMU Munich, 81377 Munich, Germany; 3 German Cancer Consortium (DKTK), Partner Site Munich, and German Cancer Research Center (DKFZ), 69120 Heidelberg, Germany; 4 Comprehensive Cancer Center Munich (CCCM), LMU Munich, 81377 Munich, Germany; 5 COVID-19 Registry of the LMU Munich (CORKUM), University Hospital, LMU Munich, 81377 Munich, Germany; 6 Department of Radiology, University Hospital, LMU Munich, 81377 Munich, Germany

**Keywords:** COVID-19, obesity, metaflammation, invasive mechanical ventilation, body composition, immunonutritional scores

## Abstract

Overweight and obesity are associated with chronic low-grade inflammation and represent risk factors for various diseases, including COVID-19. However, most published studies on COVID-19 defined obesity by the body mass index (BMI), which does not encounter adipose tissue distribution, thus neglecting immunometabolic high-risk patterns. Therefore, we comprehensively analyzed baseline anthropometry (BMI, waist-to-height-ratio (WtHR), visceral (VAT), epicardial (EAT), subcutaneous (SAT) adipose tissue masses and liver fat, inflammation markers (CRP, ferritin, interleukin-6), and immunonutritional scores (CRP-to-albumin ratio (CAR), modified Glasgow prognostic score, neutrophile-to-lymphocyte ratio, prognostic nutritional index)) in 58 consecutive COVID-19 patients of the early pandemic phase with regard to the necessity of invasive mechanical ventilation (IMV). Here, metabolically high-risk adipose tissues represented by increased VAT, liver fat, and WtHR strongly correlated with higher levels of inflammation, pathologic immunonutritional scores, and the need for IMV. In contrast, the prognostic value of BMI was inferior and absent with regard to SAT. Multivariable logistic regression analysis identified an optimized IMV risk prediction model employing liver fat, WtHR, and CAR. In summary, we suggest an immunometabolically risk-adjusted model to predict COVID-19-induced respiratory failure better than BMI-based stratification, which warrants prospective validation.

## 1. Introduction

The high prevalence and increasing incidence rates of overweight and obesity are major public health problems in Western societies due to their association with pathologies such as diabetes, cardiovascular diseases, and cancer [1,2,3,4]. On the other hand, overweight and obesity are also risk factors for a number of infectious diseases [5] and are associated with higher frequencies of nosocomial infections, including respiratory tract infections and pneumonia, as again recently discussed for COVID-19 patients [5,6,7]. Generally, an excess of visceral adipose tissue negatively affects lung functions and ventilation via mechanical and immunologic mechanisms, leading to obstructive and restrictive dysfunctions and more hypoventilated lung tissue [5,7]. Moreover, increased adipose tissue masses, especially in the visceral compartment, are associated with systemic chronic low-grade inflammation, termed metaflammation, which is maintained by a constant elevation of adipokines and cytokines, such as tumor necrosis factor alpha (TNFα) and interleukin-6 (IL-6) [7,8,9,10,11,12]. As a consequence, due to metaflammation, obese patients can suffer from ineffective immune responses without sufficient clearance of the causative pathogen [13,14].

In COVID-19 patients, these obesity-associated immunologic changes have recently been suggested to enhance hyperinflammatory immune responses [7]. However, most published studies on COVID-19 patients define obesity solely based on body mass index (BMI). In other earlier reports on patients with cardiovascular diseases, BMI was already identified as an inferior predictor of obesity-associated pathologies compared with other anthropometric measures, such as waist-to-height or -hip ratios [15]. As BMI cannot assess distinct adipose tissue distribution patterns, immunometabolic high-risk patients are thereby ill-characterized. Here, visceral adipose tissue (VAT) has been extensively described as a major contributor to metaflammation and its consecutive cardiovascular and metabolic pathologies [16,17,18]. The role of subcutaneous adipose tissue (SAT) is still controversial due to heterogeneous effects on systemic inflammation and metabolic pathologies [19]. Similar to VAT, epicardial adipose tissue (EAT) is of special interest with respect to the development of cardiovascular diseases, and its thickness has been described as a surrogate for visceral fat deposition [20]. Given EAT’s anatomical proximity to the heart, direct interactions with cardiac structures and the release of proinflammatory and fibrotic mediators can additionally drive cardiovascular pathomechanisms [20,21]. Finally, fat accumulation within the liver has been described not only as a risk factor for liver dysfunction and fibrosis but also as associated with cardiometabolic diseases [22].

Another way to depict an individual’s nutritional and inflammatory status is the use of immunonutritional scores such as the modified Glasgow prognostic score (mGPS) [23], the prognostic nutritional index (PNI) [24], and the neutrophile-to-lymphocyte ratio (NLR) [25]. These scores were originally developed to predict the outcome of cancer patients and are calculated based on serum levels of inflammatory markers, mostly including albumin as a marker of patient nutritional status and CRP reflecting the inflammatory component [26]. CRP, being part of immunonutritional scores, including mGPS and the Prognostic Index [27], was repeatedly shown to be an independent prognostic marker for COVID-19 disease severity [28,29,30]; it gradually increases with the size of pneumonic infiltrates [31] and is an inherent part of the inflammatory cascade caused by COVID-19 infection [32]. Recently, in the context of COVID-19 infections, immunonutritional scores showed a prognostic value for adverse outcomes [33]. Overall, NLR showed the highest potential as a prognostic marker for COVID-19 patients. In addition, albumin-based scores such as the CRP-to-albumin ratio (CAR) and PNI were also good predictors of COVID-19 disease severity.

In most studies, the effects of anthropometric measures, body composition, and immunonutritional scores on the outcomes of COVID-19 patients were investigated isolated from each other. Therefore, we aimed to perform a comprehensive analysis highlighting interactions within immunometabolic host factors in the context of COVID-19 disease by performing a retrospective analysis of a single-center COVID-19 cohort.

## 2. Methods

### 2.1. Patients

We performed a retrospective chart review of all patients with symptomatic confirmed COVID-19 who were hospitalized at the LMU University Hospital in Munich, Germany, between 29 February 2020 and the date of data cutoff on 6 May 2020. A confirmed case of COVID-19 was defined as a positive result on real-time reverse transcriptase polymerase chain reaction (RT-PCR) assay of nasal and pharyngeal swab specimens. Only laboratory-confirmed cases were included in the analysis. Patients without thoracic CT scans were excluded from the study. A total of 58 out of 75 patients who were admitted during this time period met these study criteria (Suppl. Figure S1). Clinical characteristics included pre-existing comorbidities (hypertension, diabetes, chronic kidney injury, chronic obstructive pulmonary disease (COPD)), laboratory parameters at the time of admission (c-reactive protein (CRP), ferritin, IL-6, albumin, differential blood count, troponin, creatinine), and patient clinical and demographic data, which were extracted from the clinical records. The overall patient cohort was split according to invasive mechanical ventilation (IMV) requirement. These criteria were chosen as analogues to previous studies assessing the severity of similar serious infectious diseases, such as H7N9 infection [34]. COVID-19 patients who did not require IMV were admitted to normal wards, where they were treated with oxygen supply via nasal cannula if needed. Within this study cohort, there was no case in which a patient was denied ICU admission due to medical circumstances or a shortage of ICU capacities. Patients are part of the COVID-19 Registry of the LMU University Hospital Munich (CORKUM, Trial ID: DRKS00021225). Patient data were anonymized for analysis, and the study was approved by the institutional review board (No: 20-767).

### 2.2. Assessment of Body Composition and Immunonutritional Scores

**Figure 1:**
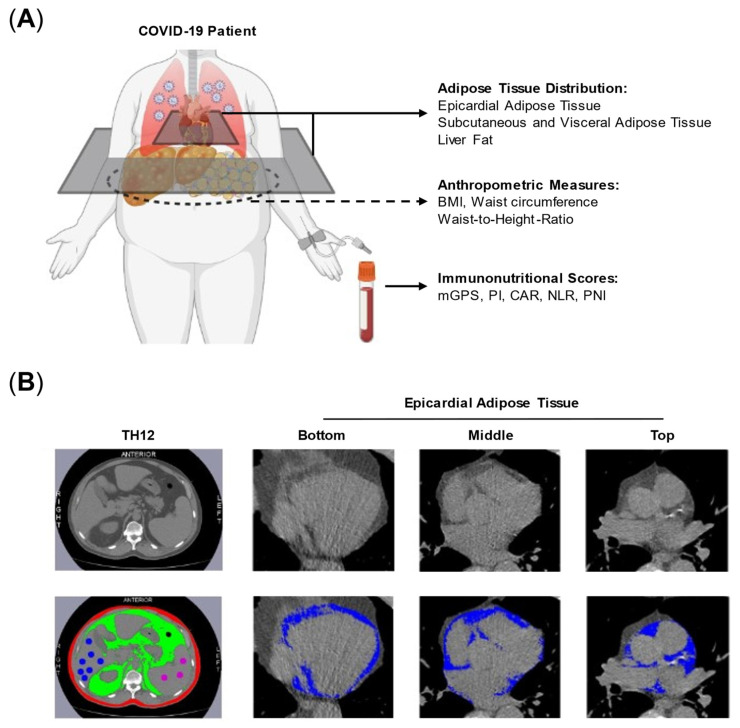
Study overview and adipose tissue quantification. (**A**) Analyses of adipose tissue distribution, anthropometric parameters and immunonutritional scores were performed on COVID-19 patients. (**B**) Examples for adipose tissue quantification at TH12 vertebrae level for visceral (green) and subcutaneous (red) adipose tissue as well as liver fat (blue spots). Epicardial adipose tissue was quantified at top, middle and bottom view of the heart (blue). Abbreviations: CAR = CRP-to-albumin ratio, mGPS = modified Glasgow Prognostic Score, NLR = Neutrophile-to-Lymphocyte-Ratio, PI = Prognostic Index, PNI = Prognostic Nutrtitional Index.

For body composition analyses, we used chest computer tomography (CT) scans taken on the day of admission or between 2 weeks before or after the diagnosis of COVID-19, depending on which was closest to the day of hospitalization. Segmentation analyses of a single CT slice at thoracic spine 12 (TH12)—as the most caudal common reference point—were performed to quantify SAT, VAT, and waist circumference as well as the fat contents of both the liver and spleen (Figure 1A,B). Adipose tissue discrimination was based on predefined Hounsfield unit (HU) ranges (−190 to −30 HU for SAT [35], −150 to −50 HU for VAT [35], and −190 to −30 HU [36] for EAT). EAT content was measured at the bottom, middle (the 4-chamber view), and top (left main coronary artery view) of the heart, and the mean of these three areas was calculated (Figure 1B). Organ fat content of the liver and spleen were determined by analysis of the HU values in randomly selected regions of interest (ROI; liver: 7 ROI; spleen: 3 ROI) in a single CT slice at TH12. A lower attenuation of the liver indicates a higher level of hepatic fat involvement. The mean HU values of the spleen were used as a control. Cross-sectional areas of respective tissues were also computed for each image. Segmentation analyses were performed Slice-O-Matic software package (version 5.0, Tomovision, Magog, Quebec, Canada). Abdominal circumference was measured with ImageJ software (version 2.0.0, U.S. National Institutes of Health, Bethesda, MD, USA).

We further calculated the following immunonutritional scores based on the laboratory values at the time point of admission: modified Glasgow prognostic score (mGPS), prognostic index (PI), prognostic nutritional index (PNI), CRP-to-albumin ratio (CAR), and neutrophile-to-lymphocyte ratio (NLR). Scoring systems and calculations are summarized in Suppl. Table S1.

### 2.3. Statistics

Patient characteristics, body composition, and serum parameter analyses were compared using the Mann–Whitney test for continuous variables and the Fisher’s exact test and Chi-squared test for categorical variables. Continuous variables are reported as median and interquartile range (IQR) if not stated otherwise. To measure the relationship between two continuous variables, Spearman correlation analyses were used. The area under the curve (AUC) and the 95% confidence interval (95% CI) of the receiver operating characteristic (ROC) analysis were computed using the predicted probability of the need for IMV. Optimal discriminatory thresholds were determined by optimizing the respective Youden J statistic. Logistic regression analyses were used to calculate univariate odds ratios (OR). Multivariable analysis was performed as both-directional stepwise binary logistic regression for the outcome of IMV requirement. The model included body composition parameters and immunonutritional scores with an AUC > 0.74 in ROC analyses. Significance was defined as *p* < 0.05. Statistical analysis was performed using GraphPad Prism v9.0 (GraphPad Software, Inc., San Diego, CA, USA) and R statistical software v4.1.0.

## 3. Results

### 3.1. Patient Characteristics

We screened the clinical records of 75 patients consecutively admitted to our medical center between February to May 2020. Based on data availability and completeness, 58 patient records were included in the analysis of anthropometric measures, body composition, and immunonutritional scores (Figure 1 and Suppl. Figure S1). Depending on the need for IMV, we subdivided the entire cohort into two groups (Table 1). All but one of the patients needed IMV due to COVID-19-associated ARDS (mild: 3, moderate: 6, severe: 5, cardiac decompensation: 1; Suppl. Table S2). The median age of the entire patient cohort was 63 years (range 32–91 years) without relevant differences between patients with IMV and without IMV. A total of 27.6% of all patients were female. A higher proportion of patients among the COVID-19 patients without IMV were female (non-IMV: 32.6% vs. IMV: 13.3%, *p* = 0.19). Within the entire patient cohort, 56.9% had none of the considered comorbidities, 31% had one, and 12% had at least a combination of two or more of the considered comorbidities. The number of comorbidities was similarly distributed between patients with and without the need for IMV (*p* = 0.39). Although diabetes was more prevalent in COVID-19 patients with IMV (IMV: 26.7% vs. non-IMV: 14%), the overall distribution of pre-existing comorbidities was similar between groups (*p* = 0.75). As laboratory surrogates for the considered pre-existing comorbidities, we compared creatinine and high-sensitive troponin levels. We found increased levels of creatinine and troponin in the serum of COVID-19 patients who needed IMV (creatinine: IMV: 1.1 (0.8–2.1) mg/dLvs. Non-IMV: 0.9 (0.4–6.0) mg/dL; troponin: IMV: 0.02 (0–0.04) ng/mL vs. non-IMV: 0 (0–0.18) ng/mL). However, median levels of creatinine were still within normal ranges (creatinine < 1.2 mg/dL), and, for troponin, barely past our institutional upper limits (troponin < 0.018 ng/mL).

### 3.2. Patients with the Need for IMV Have More Adipose Tissue and Adverse Immunonutritional Scores

Analysis of anthropometric parameters revealed a median BMI of 25.7 kg/m^2^ for the entire cohort, with increased numbers of obese patients within the IMV group (IMV: 6 (40%) vs. non-IMV: 7 (16.7%); Table 2). Accordingly, COVID-19 patients with IMV had a higher median BMI (IMV: 27.8 (20.4–45.8) kg/m^2^ vs. non-IMV: 24.8 (17.7–38.5) kg/m^2^, *p* = 0.03). Besides BMI differences, waist circumference (IMV: 111.2 (103.2–150.4) cm vs. non-IMV: 103.4 (77.7–134) cm, *p* = 0.003) and WtHR (WtHR, IMV: 0.66 (0.57–0.8) vs. non-IMV: 0.59 (0.47–0.71), *p* = 0.0006) were both significantly higher in patients who required IMV. Analysis of adipose distribution patterns from body composition analyses further displayed that the differences in anthropometric data were predominantly based on the visceral adipose depot. Here, patients who required IMV displayed a significantly higher amount of VAT compared with patients who did not need IMV (IMV: 133.4 (64.7–300.3) cm^2^ vs. non-IMV: 84.6 (7–237.2) cm^2^, *p* = 0.005), whereas SAT and EAT were increased without reaching significance level (Table 2). In addition, the IMV cohort also had higher amounts of hepatic fat as indicated by a lower attenuation in the computer tomography with significantly lower HU values (IMV: 45 (28.6–57) HU vs. non-IMV: 48.6 (31.3–61.2) HU, *p* = 0.004). As a control, we also analyzed splenic tissue attenuation, which showed no differences between the two groups (Table 2).

Next to body composition, we found significant differences in immunonutritional scores between the two cohorts. In particular, the scores including serum albumin levels showed adverse values in the IMV cohort (PNI: IMV 36.6 (27.1–46.7) vs. non-IMV 43.1 (36.4–54.8), *p* = < 0.0001; CAR: IMV 2.6 (0.6–4.9) vs. non-IMV 0.5 (0–9.8), *p* = 0.0007), whereas the scores NLR and PI, focusing on combinations of leukocyte subsets, showed a nonsignificant trend (Table 2). Results for single inflammatory markers (CRP, ferritin, interleukin-6, albumin, leukocytes) were similar and in accordance with previous publications (Suppl. Table S3).

### 3.3. ROC Analyses Identify WtHR, VAT, Liver Fat, and Immunonutritional Scores as Risk Factors for the Requirement of IMV

**Figure 2:**
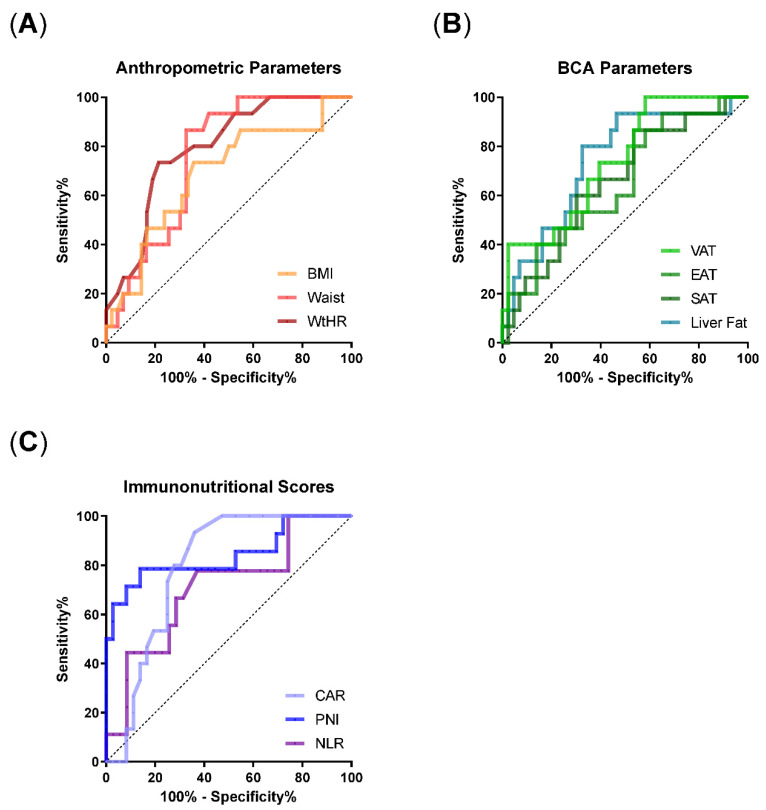
Receiver Operating Characteristic (ROC) curves for prediction of IMV need in COVID-19 patients based on anthropometric data, adipose tissue distribution and immunonutritional scores. (**A**) ROC curves for BMI, waist and WtHR. (**B**) ROC curves for liver fat, SAT, VAT and EAT. (**C**) ROC curves for CAR, PNI and NLR. Abbreviations: BMI = body mass index, CAR = CRP-to-albumin ratio, NLR = Neutrophile-to-Lymphocyte-Ratio, PNI = Prognostic Nutritional Index, Waist = waist circumference, WtHR = waist-to-height-ratio, S/V/EAT = subcutaneous/visceral/epicardial adipose tissue.

To evaluate the prognostic value of the body composition parameters and immunonutritional scores for the requirement of IMV, we performed ROC analyses (Figure 2). The scores mGPS and PI were excluded from these analyses due to their categorial distribution. Regarding the anthropometric parameters, ROC analyses revealed that waist circumference and WtHR were superior to BMI as measured by AUC (BMI: AUC 0.69 (0.53–0.85), *p* = 0.03; waist: AUC: 0.76 (0.63–0.88); WtHR: AUC 0.79 (0.67–0.91), *p* = 0.0009; Table 3). Similarly, ROC of VAT and liver fat resulted in an AUC of 0.74 (VAT: *p* = 0.006; liver fat: 0.005), whereas AUC of SAT and EAT reached maximal values of 0.66 (Table 3). Accordingly, WtHR, VAT, and liver fat showed the strongest effects in univariate logistic regression.

Regarding the immunonutritional scores, CAR (AUC 0.79 (0.67–0.91), *p* = 0.001) and PNI (AUC 0.84 (0.7–0.99), *p* = 0.0002) showed a good prognostic value for IMV requirement in ROC analyses, whereas NLR did not reach significance level. In univariate logistic regression, PNI and NLR remained prognostic factors for the requirement of IMV (Table 3). Single inflammatory parameters reached similar or better AUCs, which was particularly true for IL-6 (Suppl. Figure S2, Suppl. Table S4).

### 3.4. Metabolically High-Risk Adipose Tissue Sites Correlate with Inflammatory Parameters and Immunonutritional Scores

**Figure 3:**
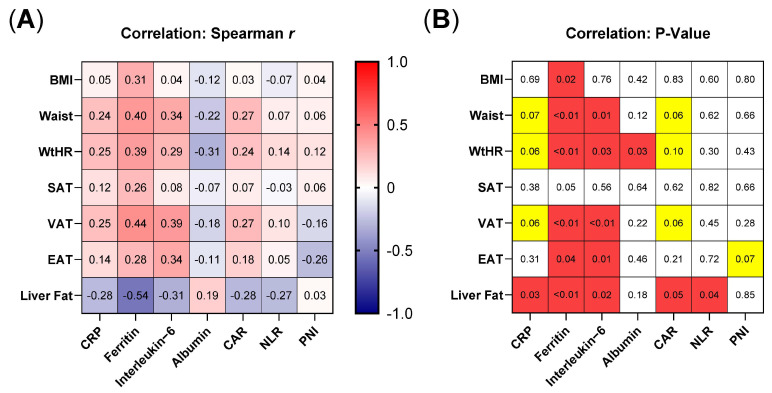
Metabolically high-risk adipose tissue compartments correlate inflammatory serum markers and immunonutritional scores. Each of the anthropometric parameters (BMI, Waist, WtHR) and adipose tissue compartments (SAT, VAT, EAT, Liver Fat) were correlated with inflammatory markers (CRP, ferritin, interleukin-6, albumin) and immunonutritional scores (CAR, NLR, PNI). (**A**) Heatmap displays Spearman correlation coefficient from -1 (blue) to 1 (red). (**B**) Matrix shows the respective p-values to correlations shown in A. Yellow cells = p-value between 0.1 and 0.05, red cells = p-value below 0.05. Abbreviations: BMI = body mass index, CAR = CRP-to-albumin ratio, NLR = Neutrophile-to-Lymphocyte-Ratio, PNI = Prognostic Nutritional Index, Waist = waist circumference, WtHR = waist-to-height-ratio, S/V/EAT = subcutaneous/visceral/epicardial adipose tissue.

To further investigate whether obesity-associated metaflammation contributes to the differences in the COVID-19 cohorts, we correlated body composition parameters with inflammatory markers and immunonutritional scores (Figure 3). We found that the body composition parameters with the biggest differences between IMV and non-IMV patients also displayed the strongest correlations with inflammatory markers and immunonutritional scores. For instance, among the anthropometric parameters, WtHR significantly correlated with ferritin, IL-6, and albumin levels (WtHR/ferritin: r = 0.39, *p* = < 0.01; WtHR/IL-6: r = 0.29, *p* = 0.03; WtHR/albumin: r = −0.31, *p* = 0.03) and showed correlation trends with CRP and CAR. In contrast, BMI only correlated with ferritin (BMI/ferritin: r = 0.31, *p* = 0.02). Regarding adipose tissue distribution, similar correlations were noticed for VAT and liver fat, with the latter displaying the strongest effects overall. Notably, SAT did not show any correlations or trends. Overall, correlations were generally stronger for CRP, ferritin, and IL-6 compared with albumin and were stronger between body composition parameters with single inflammatory markers compared to immunonutritional scores.

### 3.5. Stepwise Multivariable Logistic Regression Identifies an Optimal Model for IMV Requirement including Liver Fat, WtHR, and CAR

Since body composition parameters correlated with inflammatory markers and immunonutritional scores, we next wanted to evaluate these parameters in a multivariable model for the prediction of IMV requirement. For the base model, we included all parameters with an AUC > 0.74 based on the ROC results, and we dichotomized variables based on discriminatory thresholds calculated by Youden statistics (Table 3). Due to multicollinearity of waist circumference and WtHR, only WtHR was retained in the base model, which finally included the parameters WtHR, VAT, liver fat, CAR, and PNI. Because of the small study cohort, we performed a stepwise both-directional multivariable logistic regression analysis resulting in a final model that included liver fat (OR 5.6 (1.03–38.3), *p* = 0.02), WtHR (OR 5.6 (1.11–35.5), *p* = 0.07), and the immunonutritional score CAR (OR 22.3 (3–496.1), *p* = 0.03) (Table 4). Thus, in our modeling approach, body composition parameters and immunonutritional scores are independently associated with IMV requirement in COVID-19 patients.

## 4. Discussion

Obesity has been described as a risk factor for developing severe COVID-19 and respiratory failure. However, most published studies employed only BMI-based determination of overweight and obesity. In this study, we aimed to perform a comprehensive analysis of immunometabolic host features, including body composition and laboratory-based inflammation and nutrition status, with regard to their impact on the risk for respiratory failure and invasive ventilation in hospitalized COVID-19 patients of the early pandemic phase. Our results suggest that the assessment of immunometabolically active—especially visceral—adipose tissue sites is superior to the widely used BMI, which neglects body composition and tissue distribution patterns. Moreover, in this cohort, we demonstrate that the combination of adipose tissue quantification with immunonutritional scores based on standard laboratory data added further value to identify patients at the highest risk for the need for IMV.

We found increased waist circumference, WtHR, VAT mass, and liver fat contents to be strongly associated with IMV requirement. From the anthropometric measures, an increased waist circumference or WtHR was a stronger predictor for IMV than a BMI-based assessment of obesity. This is in line with data from the pre-COVID-19 era. Here, a large meta-analysis demonstrated the superiority of WtHR compared with BMI in assessing the risk for cardiometabolic diseases [15]. In addition, waist circumference as a measure of visceral obesity was also associated with higher risks of influenza in adults and children compared with BMI measures [37,38]. Similarly, in our cohort, adipose tissue distribution patterns confirmed the high immunometabolic risk derived from visceral fat depots compared with subcutaneous and epicardial adipose tissue sites, which is displayed by the increased risk of COVID-19 patients for IMV. Previous reports on COVID-19 patients have shown similar results [39,40,41,42,43]. In addition to the published literature on body composition analyses in COVID-19 patients, this study additionally investigated CT-derived liver fat content based on radiologic signal attenuation within the liver. With this approach, we found significantly lower liver signals in the IMV cohort, indicating a higher liver fat involvement in those patients. An increased liver fat content was a strong prognostic factor for IMV risk and comparable to visceral adipose tissue mass. As a control, spleen attenuation was neither significantly associated with invasive mechanical ventilation nor did it correlate with laboratory inflammation indicators. The strong effects exerted by liver fat in our cohort might reflect the transformation from a metabolically low-risk state to a metabolically high-risk state of obesity, as discussed previously [44], and matches the findings of other cohorts, in which nonalcoholic/metabolic associated fatty liver disease (N/MAFLD) is associated with a worse outcome for COVID-19 patients [45]. However, whether MAFLD displays an independent risk factor or is rather a byproduct of visceral obesity remains controversial [46,47,48]. To conclude, our study adds further information that overweight and obesity play a crucial role in COVID-19 and highlights that the widely accepted single use of BMI as a measure of excess adipose tissue is insufficient and might not display the effective risk.

To comprehensively assess the immunometabolic status of the present cohort, we complemented body composition analyses with calculations of immunonutritional scores. Here, we employed established scores that include serum albumin levels in combination with several immunological parameters, such as CRP, lymphocyte, or neutrophil numbers. Originally developed as prognostic scores in solid cancer patients, the use of these scores was also validated in other diseases, such as postoperative infections [49,50,51,52], cardiovascular diseases [53] and rheumatologic diseases [54,55,56]. Particularly in the context of COVID-19, NLR, CAR, and PNI have been described as prognostic markers for disease severity and survival. In our cohort, mGPS, PNI, and CAR had the strongest correlation with IMV needs, whereas PI and NLR were less strongly associated. This strengthens previous data that found albumin-based immunonutritional scores such as PNI have a high prognostic value for COVID-19 disease severity [33,57,58]. Taken together, immunonutritional scores seem to reflect an adverse metabolic and inflammatory status in COVID-19 patients, but more studies are needed to clarify whether their use is superior to the single factors they are composed of.

Since the beginning of the COVID-19 pandemic, several studies have analyzed potential underlying mechanisms mediating obesity-associated risks for COVID-19 patients. In this regard, the following mechanisms were discussed: an adipocyte-associated increase in thrombogenic material within the blood, pulmonary microvascular dysfunction, and functional impairment of the alveolar-capillary unit mediated by obesity-associated lung inflammation and obesity-associated impaired lung physiology [59]. Moreover, the interaction of SARS-COV-2 with the renin–angiotensin system (RAAS) via angiotensin-converting enzyme 2 (ACE2) has attracted widespread attention as a mechanism [60,61]. SARS-COV-2 predominantly binds to ACE2-expressing tissues, which leads to a disbalance in the RAAS cascade, resulting in increased cytokine release [60]. Since adipose tissue abundantly expresses ACE2 [62,63], it was hypothesized that it might serve as a viral reservoir enabling viral shedding, immune activation, and cytokine amplification, predisposing obese COVID-19 patients to a severe course of the disease [60]. However, data on obesity-associated ACE2 expression patterns are still in part conflicting; thus, further studies are needed in this regard [64]. Moreover, the role of adipokines was evaluated in the context of COVID-19, showing that adiponectin as an anti-inflammatory cytokine is decreased in COVID-19 patients [65]. Regarding the proinflammatory adipokine leptin, results are yet inconclusive [66,67]. In addition to these mechanisms, obesity-associated low-grade inflammation (i.e., metaflammation) was suspected of contributing to worse outcomes in overweight and obese patients [7,59,68]. To further investigate metaflammation as a mechanism, we correlated body composition parameters with inflammatory markers and immunonutritional scores. Notably, among the quantified adipose tissue sites, known metabolically active deposits like VAT and liver fat showed strong correlations with inflammatory markers such as IL-6, ferritin, and albumin in our cohort, whereas the metabolically rather inactive SAT did not correlate with any of the inflammatory markers. Similarly, anthropometric features reflecting visceral obesity such as waist and WtHR showed stronger correlations than BMI. The association between increased VAT/MAFLD measures and chronic inflammation has already been well described [69,70] as a consequence of adipose tissue infiltrating activated immune cells [71]. Although these clinical analyses are not sufficient to dissect mechanistic causalities, our findings point toward an active part of metabolically high-risk adipose tissue sites for systemic inflammatory processes and add further evidence for the role of metaflammation in COVID-19 and infectious diseases.

Limitations of this study include its retrospective and single-center design, as well as the overall small sample size and the differences between group sizes. In addition, subgroup analyses of male and female patients that would have been of high interest in the light of sex-specific body composition patterns and different degrees of metaflammation could not be performed due to the fact that males were the dominating cohort. Various differences in innate and adaptive immune responses between men and women have already been described. As an example, in women, macrophages and neutrophils show higher phagocytic and degranulation activities, dendritic cells present antigens to T cells more efficiently, and B cells are more abundant compared to men. In contrast, men have more natural killer cells, and upon stimulation, CD4-positive T cells more often mount an IL-17-polarized response compared with TH1-type and interferon-γ polarized responses in women [72]. In addition, adipose distribution patterns also differ between males and females, with males tending to develop higher amounts of VAT, whereas, in women, subcutaneous adipose tissue plays an important role [16]. Since sex impacts both immune system functionality and adipose tissue distribution patterns, larger patient cohorts are needed to evaluate the impact of sex disparities on the proposed immunometabolically risk-adapted model for COVID-19 outcomes. Finally, since our cohort represents the early pandemic phase and the patients were presumably infected with the SARS-CoV-2 wild-type strain only, our results need to be validated for later SARS-CoV-2 variants that are characterized by specific mutations within the viral core and spike proteins, which impact their virulence and immunogenicity [73,74].

Still, these present findings are hypothesis-generating, and further clinical and translational investigation is warranted. If confirmed prospectively, we see useful clinical applications. First, our results highlight the potential impact of overweight and obesity on infectious diseases, which becomes more important as incidence rates of overweight and obesity in (emerging) industrialized countries are rapidly increasing [75]. This is specifically important in the context of the simultaneously increasing distribution of resistant pathogens [76]. Therefore, public diet- and exercise-based interventions might be useful to not only reduce the risk of cardiovascular diseases and cancer but also to diminish the risk of developing severe infectious diseases, finally leading to lower hospitalization rates and public health costs. Second, the present study shows that routinely performed thoracic CT scans in respiratory diseases might comprise additional diagnostic value for the assessment of body composition. Since CT scans can be used to directly measure adipose tissue areas or volumes, it is considered a reference method for the quantification of adipose deposits. In comparison to magnetic resonance imaging, CT-based quantification is less likely to be affected by breathing artifacts [77]. Whereas body composition is typically assessed on the L3 vertebrae level [77], we implemented an analysis algorithm measuring on the level of the 12th thoracic spine as the most common caudal spine of the present cohort. Similar analyses have been described as robust and valid direct measurements of adipose tissue in patients after lung transplantation [78]. Thus, in patients with respiratory infections, body composition might be easily assessed by low-dose CT scans at TH12 to evaluate metabolically high-risk adipose tissue sites without the need for further diagnostic procedures.

## 5. Conclusions

The present study suggests that CT-derived assessments of anthropometric measures such as WtHR and of metabolically high-risk adipose tissue distributions including liver fat in combination with immunonutritional scores are superior to BMI in predicting the necessity for IMV in COVID-19 patients. Body composition measurements can easily be performed by segmentation analysis of caudal thoracic CT images and do not require additional diagnostic procedures or larger CT areas. Moreover, the link between metabolically high-risk adipose tissue compartments, immunonutritional scores, and inflammatory markers indicate that obesity-associated metaflammation might play a critical role in SARS-CoV-2-triggered hyperinflammatory responses and ARDS development.

## Figures and Tables

**Table 1:** Baseline Patient Characteristics.

Characteristic		Invasive Mechanical Ventilation		*p*-Values
	All Patients (N = 58)	No (N = 43)	Yes (N = 15)	
**Age, median (range) [years]**	63 (32–91)	61 (31–91)	64 (47–82)	0.66
30–50 years	13 (22.4)	12 (27.9)	1 (6.7)	0.13
51–70 years	27 (46.6)	17 (39.5)	10 (66.7)	
>71 years	18 (31)	14 (32.6)	4 (26.7)	
**Female**	16 (27.6)	14 (32.6)	2 (13.3)	0.19
**Comorbidities**				
None	33 (56.9)	24 (55.8)	9 (60)	0.39
1 comorbidity	18 (31)	15 (34.9)	3 (20)	
≥2 comorbidities	7 (12)	4 (9.3)	3 (20)	
Diabetes	10 (27.2)	6 (14)	4 (26.7)	0.75
Coronary heart disease	13 (22.4)	10 (23.3)	3 (20)	
COPD	5 (8.6)	4 (9.3)	1 (6.7)	
Chronic kidney disease	5 (8.6)	4 (9.3)	1 (6.7)	
**Serum parameters**				
Creatinine, median (range) [mg/dL]	0.95 (0.4–6.0)	0.9 (0.4–6.0)	1.1 (0.8–2.1)	0.006
Troponin, median (range) [ng/mL]	0 (0–0.18)	0 (0–0.18)	0.02 (0–0.04)	0.002

All values are shown in number (percent) if not stated otherwise. Abbreviations: COPD = chronic obstructive pulmonary disease.

**Table 2:** Distribution of Anthropometric and Body Composition Parameters and Immunonutritional Scores Between COVID-19 Cohorts Based on IMV Requirement.

	Invasive Mechanical Ventilation			
Characteristic	All Patients (N = 58)	No (N = 43)	Yes (N = 15)	*p*-Values
**Anthropometric Parameters**				
BMI [kg/m²]	25.7 (17.7–45.8)	24.8 (17.7–38.5)	27.8 (20.4–45.8)	0.03
BMI ≥ 30, number (percent) [kg/m²]	13 (22.8%)	7 (16.7%)	6 (40%)	
Waist circumference [cm]	107.5 (77.7–150.4)	103.4 (77.7–134)	111.2 (103.2–150.4)	0.003
WtHR [rel.]	0.61 (0.47–0.8)	0.59 (0.47–0.71)	0.66 (0.57–0.8)	0.0006
**Adipose Tissue Distribution**				
SAT [cm²]	97 (8.5–383.6)	92.9 (8.5–383.6)	118 (40.8–343.7)	0.07
VAT [cm²]	88.9 (7–300.3)	84.6 (7–237.2)	133.4 (64.7–300.3)	0.005
EAT [cm²]	12.3 (3.4–32.3)	11.9 (3.4–30.7)	13.2 (5.9–32.3)	0.08
Liver Fat [HU]	46.7 (28.6–61.2)	48.6 (31.3–61.2)	45 (28.6–57)	0.0044
Spleen [HU]	44.4 (29–55.1)	44.4 (29–55.1)	45.7 (31.2–54.8)	0.984
**Immunonutritional Scores**				
NLR [rel.]	4.3 (0.9–20.4)	3.5 (0.9–19.1)	5.8 (2.5–20.4)	0.06
PNI [rel.]	42.6 (27.1–54.8)	43.1 (36.4–54.8)	36.6 (27.1–46.7)	<0.0001
CAR [rel.]	0.8 (0–9.8)	0.5 (0–9.8)	2.6 (0.6–4.9)	0.0007
mGPS				
– 0	45	35	10	0.007
– 1	6	6	0	
– 2	7	2	5	
PI				
– 0	43	35	8	0.09
– 1	12	6	6	
– 2	3	2	1	

Abbreviations: BMI = body mass index, CAR = CRP-to-albumin ratio, mGPS = modified Glasgow Prognostic Score, NLR = Neutrophile-to-Lymphocyte-Ratio, PI = Prognostic Index, PNI = Prognostic Nutritional Index, Waist = waist circumference, WtHR = waist-to-height-ratio, S/V/EAT = subcutaneous/visceral/epicardial adipose tissue.

**Table 3:** Results of ROC Analyses and Odds Ratios for Anthropometric Parameters, Adipose Tissue Distribution and Immunonutritional Scores.

	AUC (95%CI)	*p* Value AUC	Discriminatory Value	OR (95%CI)	*p*-Value OR
**Anthropometric Parameters**					
BMI	0.69 (0.53–0.85)	0.03	26.1 kg/m²	1.13 (1.01–1.29)	0.04
Waist	0.76 (0.63–0.88)	0.003	109.3 cm	1.09 (1.03–1.16)	0.009
WtHR	0.79 (0.67–0.91)	0.0009	0.635 cm/m²	1.21 (1.09–1.4)	0.002
**Adipose Tissue Distribution**					
SAT	0.66 (0.5– 0.82)	0.07	86.7 cm²	1.01 (1–1.01)	0.16
VAT	0.74 (0.6–0.88)	0.006	67.4 cm²	1.01 (1.01–1.02)	0.006
EAT	0.65 (0.49–0.81)	0.08	9.7 cm²	1.09 (1–1.2)	0.048
Liver fat	0.74 (0.6–0.89)	0.005	46.2 HU	0.88 (0.79–0.97)	0.01
**Inflammation Scores**					
CAR	0.79 (0.67–0.91)	0.001	0.7	1.28 (0.97–1.76)	0.1
PNI	0.84 (0.7–0.99)	0.0002	38.7	1.15 (1.02–1.32)	0.03
NLR	0.71 (0.51–0.9)	0.057	4.75	1.17 (1.05–1.43)	0.01

Abbreviations: 95% CI = 95% confidence interval, AUC = area under the curve, BMI = body mass index, CAR = CRP-to-albumin ratio, HU = Hounsfield unit, NLR = Neutrophile-to-Lymphocyte-Ratio, OR = Odds Ratio, PNI = Prognostic Nutritional Index, Waist = waist circumference, WtHR = waist-to-height-ratio, S/V/EAT = subcutaneous/visceral/epicardial adipose tissue.

**Table 4:** Step-wise Multivariable Logistic Regression Analysis Identifies an Optimal Model Including Liver Fat, WtHR and CAR for the Prediction of IMV Requirement.

Parameter	Discriminatory Threshold	Odds Ratio	95%CI	*p*-Value
Liver Fat	< 46.2 HU	5.6	1.03–38.3	0.02
WtHR	> 0.635	5.6	1.11–35.5	0.07
CAR	> 0.7	22.3	3.01–496.1	0.03

Abbreviations: 95%CI = 95% confidence interval, CAR = CRP-to-albumin ratio, HU = Hounsfield unit, WtHR = Waist-to-Height-Ratio.

## Data Availability

The data presented in this study are available on request from the corresponding author.

## Supplementary Materials

The following are available online at https://www.mdpi.com/article/10.3390/nu14204280/s1, Figure S1: Patient selection. Figure S2: ROC analyses of inflammatory markers for the prediction of invasive mechanical ventilation in COVID-19 patients; Table S1: Composition of Nutritional Inflammation Scores; Table S2: Additional Patient Characteristics; Table S3: Distribution of Inflammatory Markers Between COVID-19 Cohorts Based on IMV Requirement; Table S4: Results of ROC Analyses and Odds Ratios of Inflammatory Markers.
