# Supplementary material for: Risk Stratification Based on a Pattern of Immunometabolic Host Factors Is Superior to Body Mass Index—Based Prediction of COVID-19-Associated Respiratory Failure
Source: Nutrients. 2022 Oct 13;14(20):4280. doi: 10.3390/nu14204280 (PMC9611334; doi:10.3390/nu14204280)
Supplement: Supplementary file 1 [file nutrients-14-04280-s001.zip › nutrients-1968039-supplementary.pptx]

## Slide 1
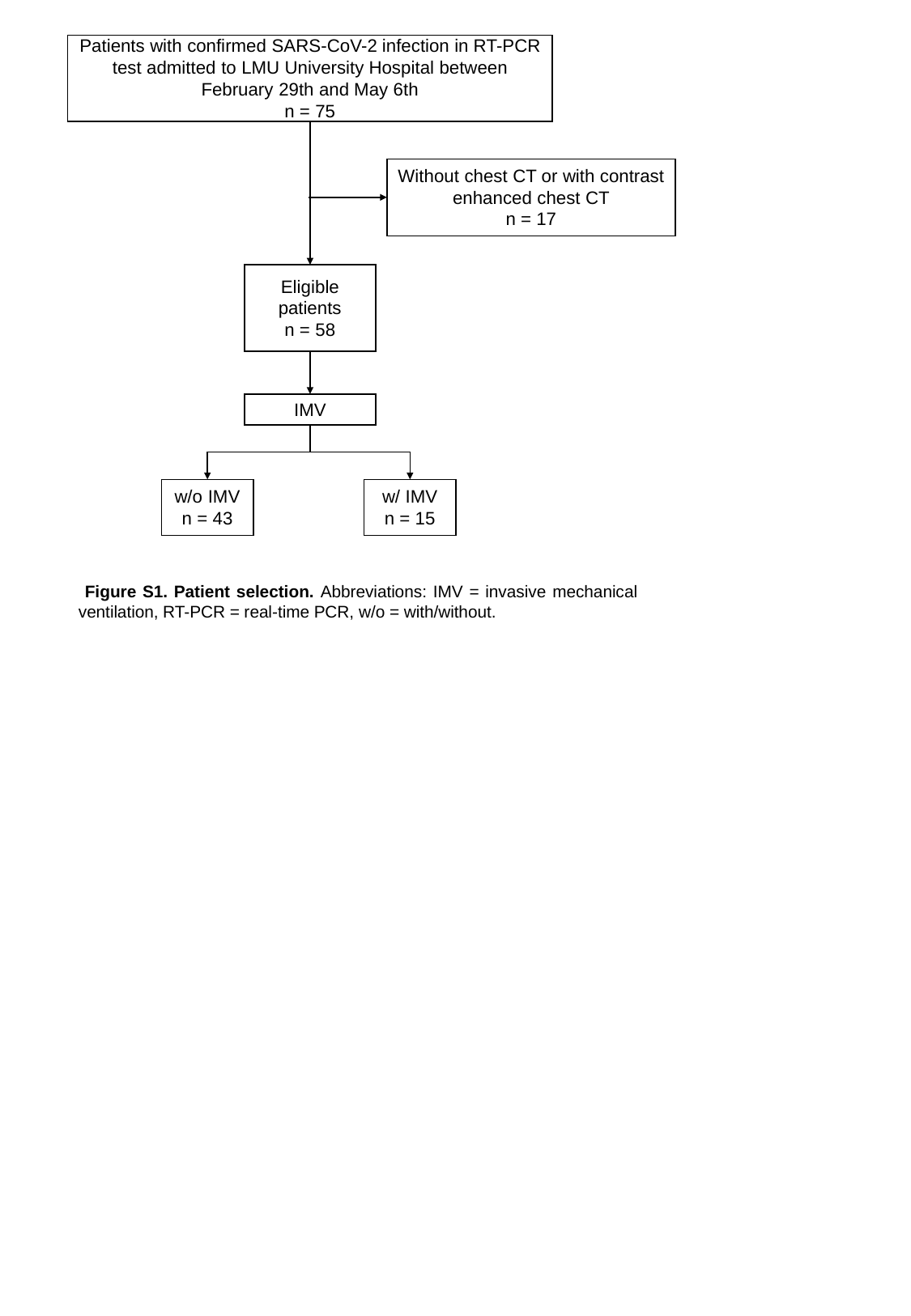

Figure S1. Patient selection. Abbreviations: IMV = invasive mechanical ventilation, RT-PCR = real-time PCR, w/o = with/without.

## Slide 2
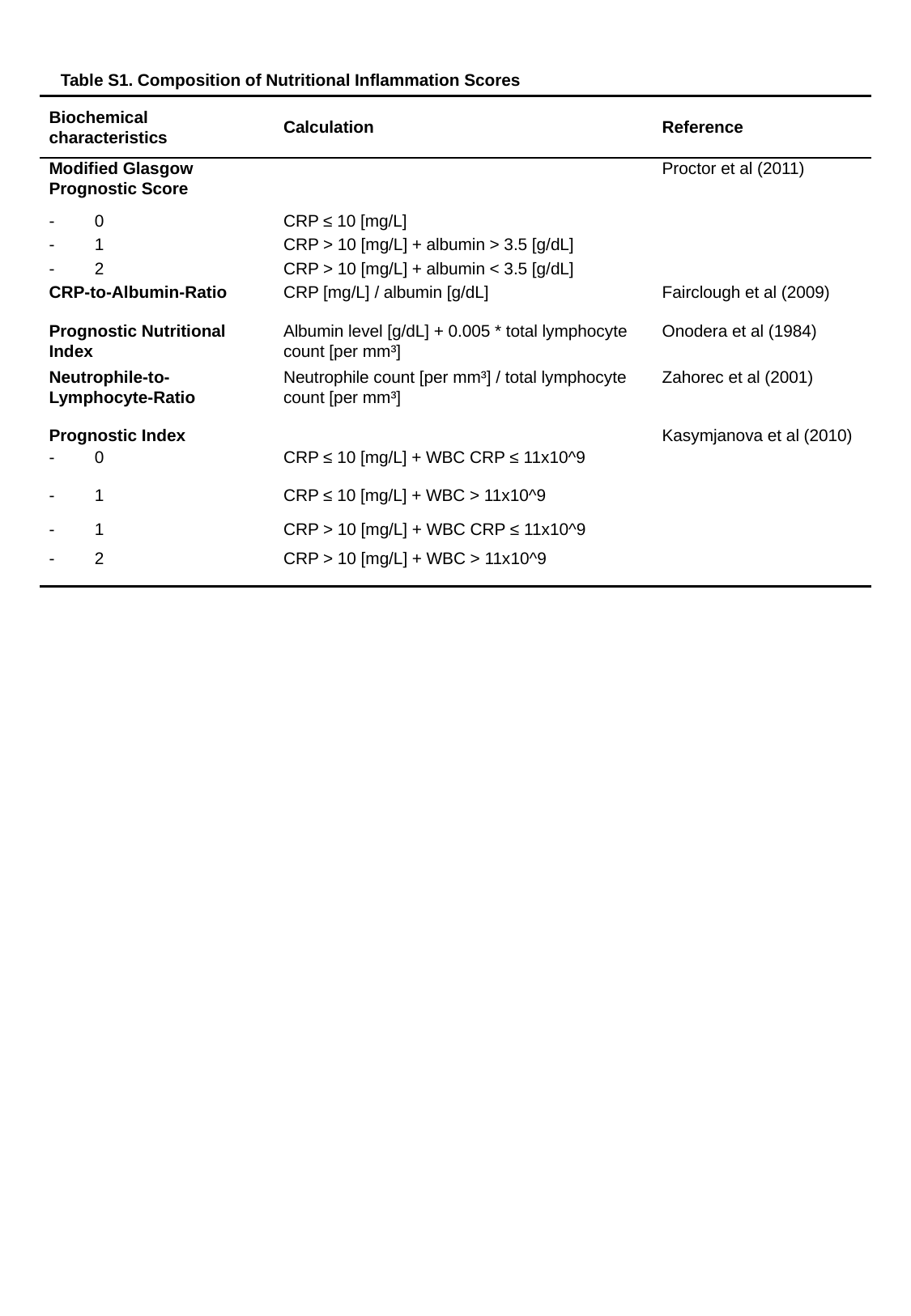

Table S1. Composition of Nutritional Inflammation Scores
| Biochemical characteristics | Calculation | Reference |
| --- | --- | --- |
| Modified Glasgow Prognostic Score | | Proctor et al (2011) |
| 0 | CRP ≤ 10 [mg/L] | |
| 1 | CRP > 10 [mg/L] + albumin > 3.5 [g/dL] | |
| 2 | CRP > 10 [mg/L] + albumin < 3.5 [g/dL] | |
| CRP-to-Albumin-Ratio | CRP [mg/L] / albumin [g/dL] | Fairclough et al (2009) |
| Prognostic Nutritional Index | Albumin level [g/dL] + 0.005 \* total lymphocyte count [per mm³] | Onodera et al (1984) |
| Neutrophile-to-Lymphocyte-Ratio | Neutrophile count [per mm³] / total lymphocyte count [per mm³] | Zahorec et al (2001) |
| Prognostic Index | | Kasymjanova et al (2010) |
| 0 | CRP ≤ 10 [mg/L] + WBC CRP ≤ 11x10^9 | |
| 1 | CRP ≤ 10 [mg/L] + WBC > 11x10^9 | |
| 1 | CRP > 10 [mg/L] + WBC CRP ≤ 11x10^9 | |
| 2 | CRP > 10 [mg/L] + WBC > 11x10^9 | |

## Slide 3
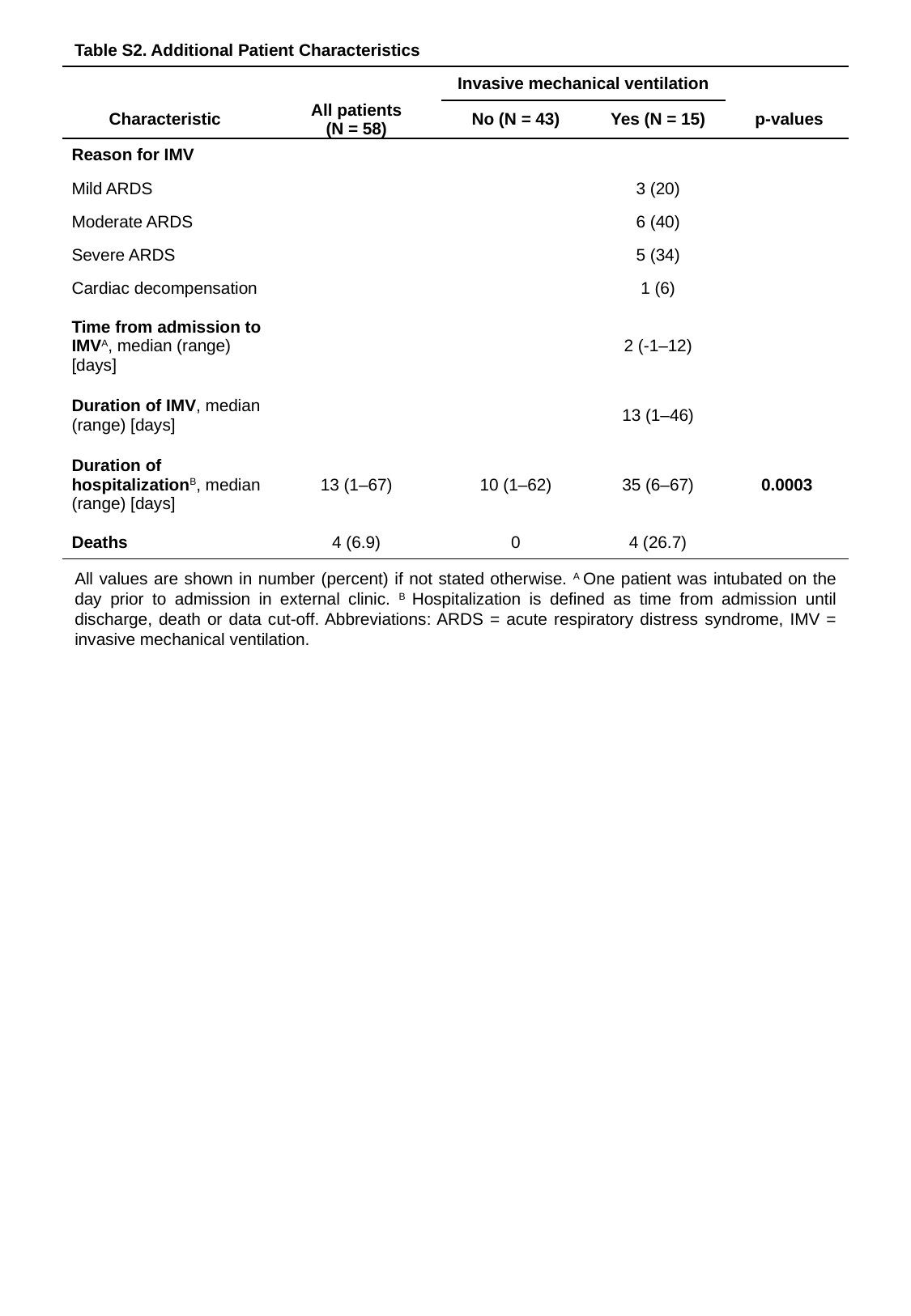

Table S2. Additional Patient Characteristics
| | | Invasive mechanical ventilation | | |
| --- | --- | --- | --- | --- |
| Characteristic | All patients (N = 58) | No (N = 43) | Yes (N = 15) | p-values |
| Reason for IMV | | | | |
| Mild ARDS | | | 3 (20) | |
| Moderate ARDS | | | 6 (40) | |
| Severe ARDS | | | 5 (34) | |
| Cardiac decompensation | | | 1 (6) | |
| Time from admission to IMVA, median (range) [days] | | | 2 (-1–12) | |
| Duration of IMV, median (range) [days] | | | 13 (1–46) | |
| Duration of hospitalizationB, median (range) [days] | 13 (1–67) | 10 (1–62) | 35 (6–67) | 0.0003 |
| Deaths | 4 (6.9) | 0 | 4 (26.7) | |
All values are shown in number (percent) if not stated otherwise. A One patient was intubated on the day prior to admission in external clinic. B Hospitalization is defined as time from admission until discharge, death or data cut-off. Abbreviations: ARDS = acute respiratory distress syndrome, IMV = invasive mechanical ventilation.

## Slide 4
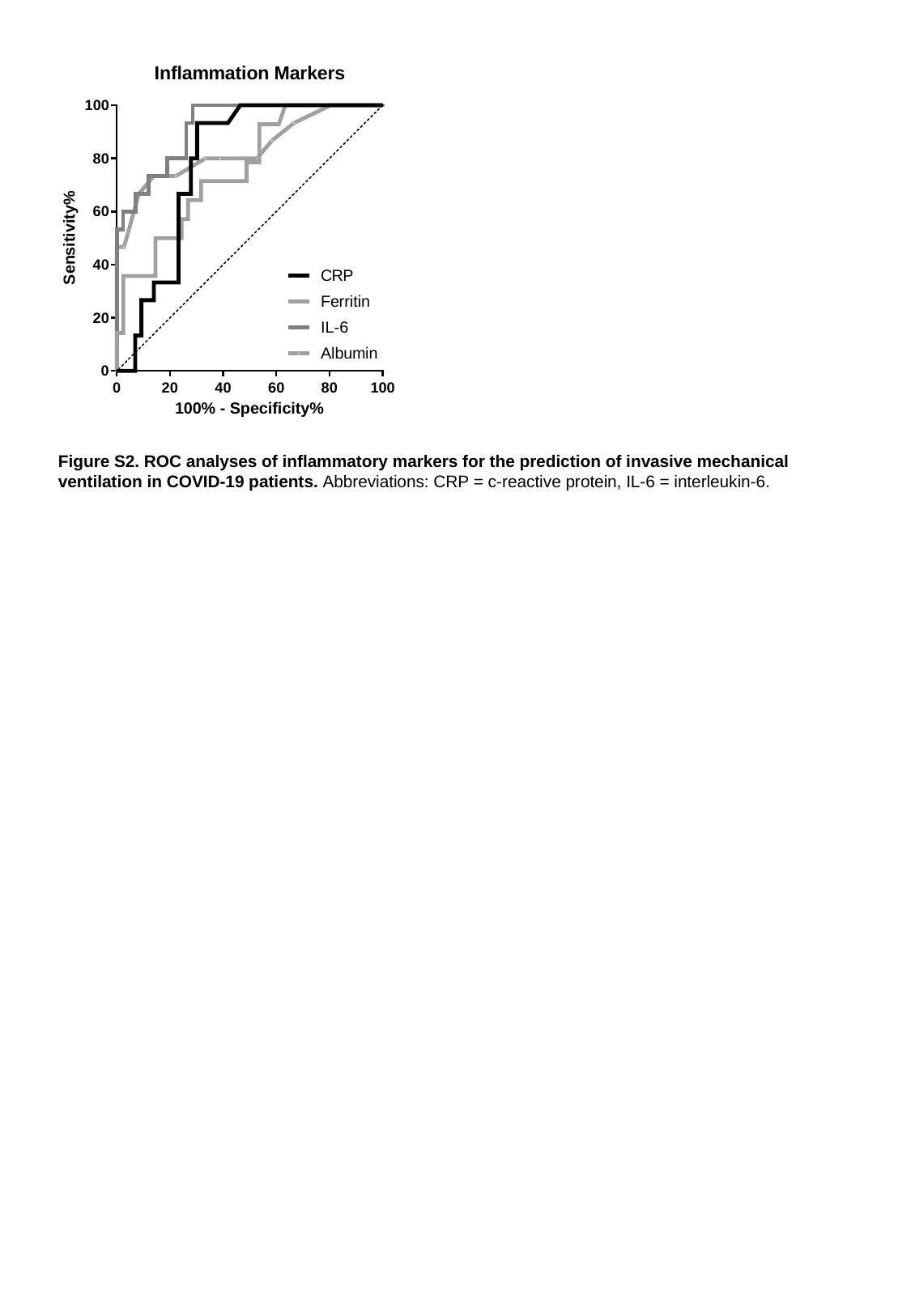

Figure S2. ROC analyses of inflammatory markers for the prediction of invasive mechanical ventilation in COVID-19 patients. Abbreviations: CRP = c-reactive protein, IL-6 = interleukin-6.

## Slide 5
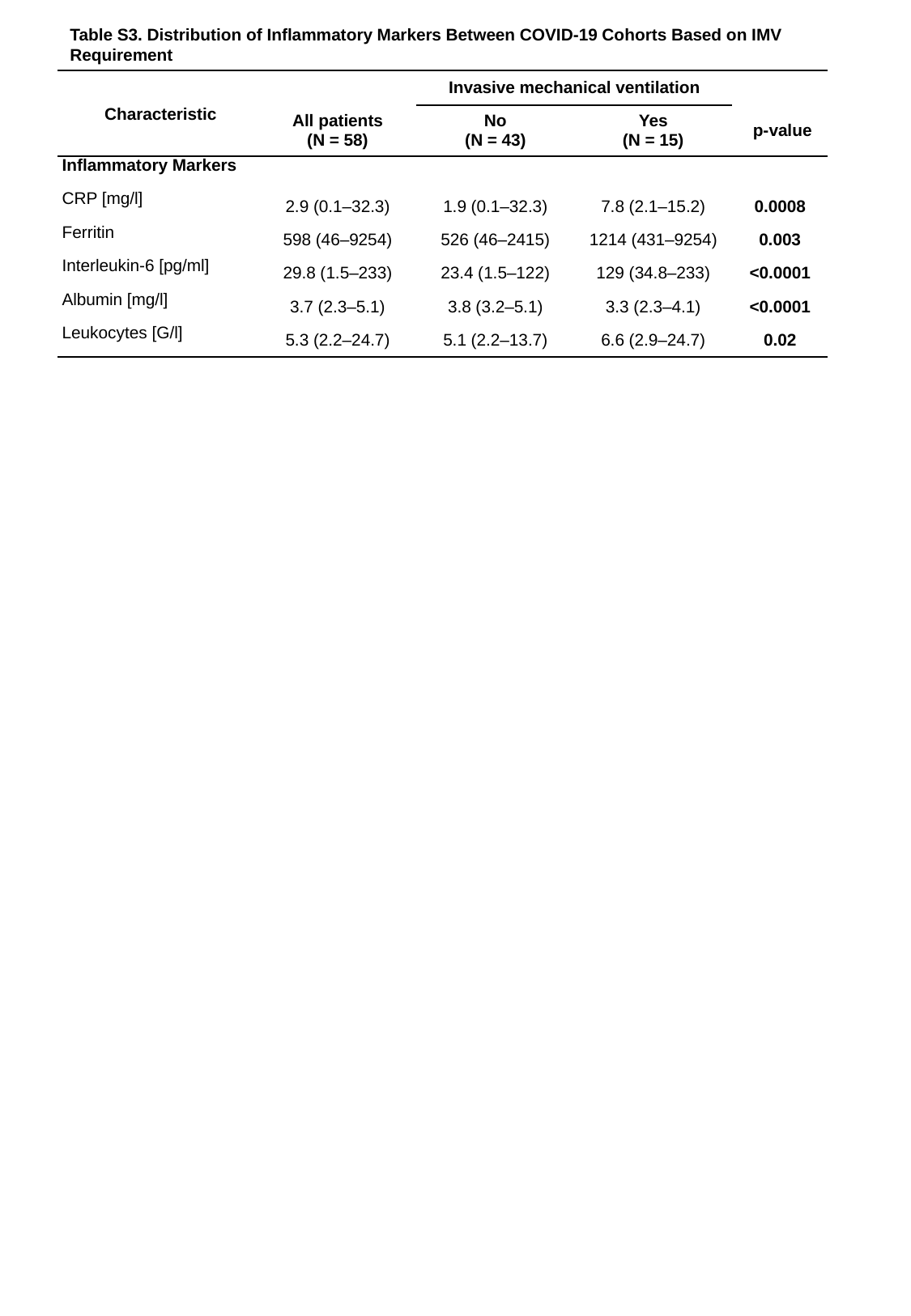

Table S3. Distribution of Inflammatory Markers Between COVID-19 Cohorts Based on IMV Requirement
| | | Invasive mechanical ventilation | | |
| --- | --- | --- | --- | --- |
| Characteristic | All patients (N = 58) | No (N = 43) | Yes (N = 15) | p-value |
| Inflammatory Markers | | | | |
| CRP [mg/l] | 2.9 (0.1–32.3) | 1.9 (0.1–32.3) | 7.8 (2.1–15.2) | 0.0008 |
| Ferritin | 598 (46–9254) | 526 (46–2415) | 1214 (431–9254) | 0.003 |
| Interleukin-6 [pg/ml] | 29.8 (1.5–233) | 23.4 (1.5–122) | 129 (34.8–233) | <0.0001 |
| Albumin [mg/l] | 3.7 (2.3–5.1) | 3.8 (3.2–5.1) | 3.3 (2.3–4.1) | <0.0001 |
| Leukocytes [G/l] | 5.3 (2.2–24.7) | 5.1 (2.2–13.7) | 6.6 (2.9–24.7) | 0.02 |

## Slide 6
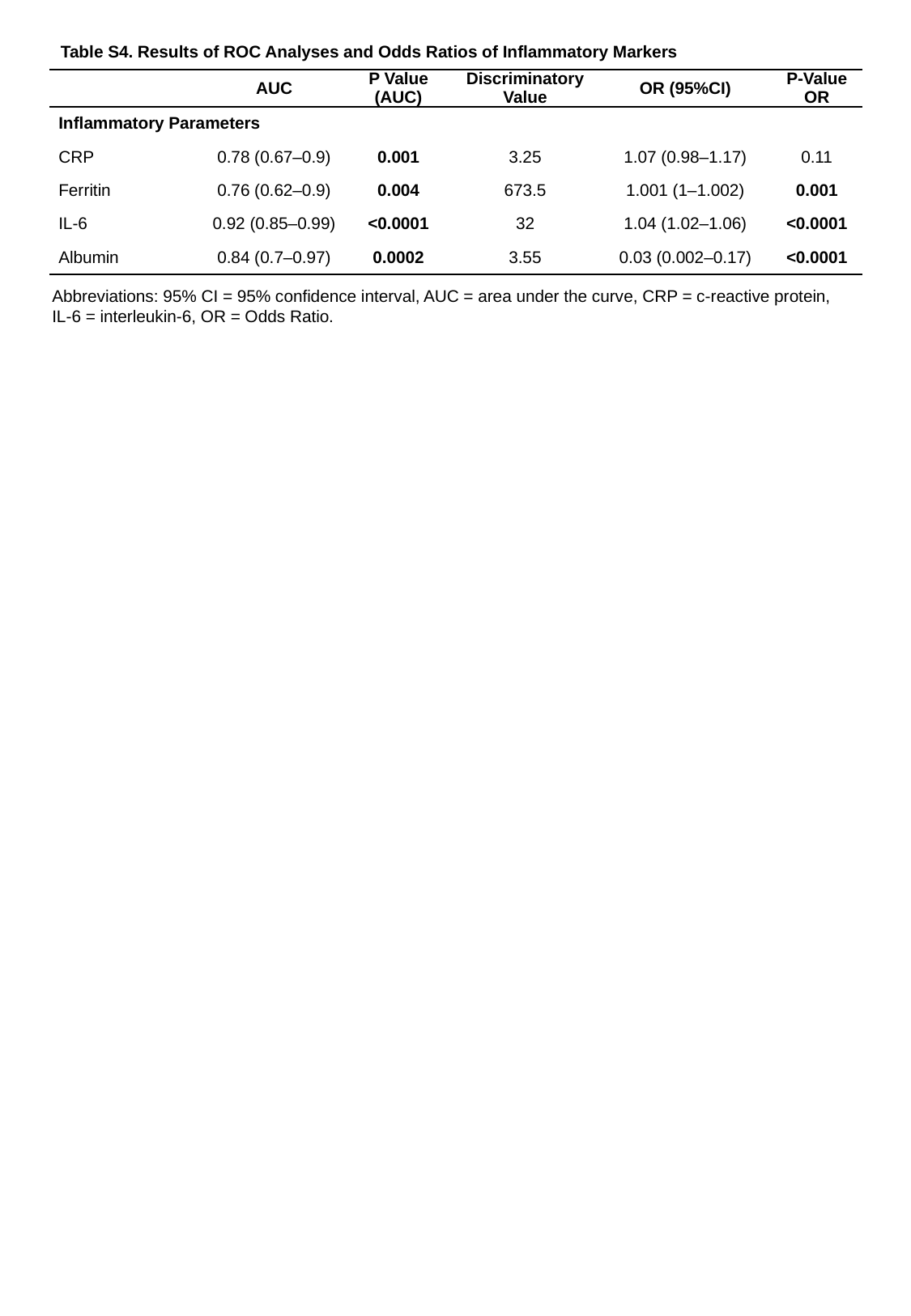

Table S4. Results of ROC Analyses and Odds Ratios of Inflammatory Markers
| | AUC | P Value (AUC) | Discriminatory Value | OR (95%CI) | P-Value OR |
| --- | --- | --- | --- | --- | --- |
| Inflammatory Parameters | | | | | |
| CRP | 0.78 (0.67–0.9) | 0.001 | 3.25 | 1.07 (0.98–1.17) | 0.11 |
| Ferritin | 0.76 (0.62–0.9) | 0.004 | 673.5 | 1.001 (1–1.002) | 0.001 |
| IL-6 | 0.92 (0.85–0.99) | <0.0001 | 32 | 1.04 (1.02–1.06) | <0.0001 |
| Albumin | 0.84 (0.7–0.97) | 0.0002 | 3.55 | 0.03 (0.002–0.17) | <0.0001 |
Abbreviations: 95% CI = 95% confidence interval, AUC = area under the curve, CRP = c-reactive protein, IL-6 = interleukin-6, OR = Odds Ratio.
